# A proposal of a fecal scoring system based on physicochemical analyses of suckling pig feces

**DOI:** 10.1186/s40813-024-00417-2

**Published:** 2025-01-09

**Authors:** Ana Maria Pons, Blanca González, Joan Pujols, Jordi Serratosa, Joaquim Segalés, Ernesto A. Gómez, Jorge Martínez

**Affiliations:** 1 Mevet SAU – Vall Companys Group, 25191 Lleida, Spain; 2 Departament de Sanitat i Anatomia Animals, Facultat de Veterinària, Campus de la Universitat Autònoma de Barcelona (UAB), 08193 Bellaterra, Barcelona Spain; 3 CEGECO –Vall Companys Group, 25191 Lleida, Spain; 4 IRTA, Animal Health, Centre de Recerca en Sanitat Animal (CReSA), Campus de la Universitat Autònoma de Barcelona (UAB), 08193 Bellaterra, Catalonia Spain; 5 Unitat Mixta d’Investigació IRTA-UAB en Sanitat Animal, Centre de Recerca en Sanitat Animal (CReSA), Campus de la Universitat Autònoma de Barcelona (UAB), 08193 Bellaterra, Catalonia Spain; 6 WOAH Collaborating Centre for Emerging and Re-Emerging Pig Diseases in Europe, IRTA-CReSA, 08193 Bellaterra, Barcelona Spain; 7 Departament de Psiquiatria i Medicina Legal de la Facultat de Medicina, Campus de la Universitat Autònoma de Barcelona (UAB), 08193 Bellaterra, Catalonia Spain; 8 Centro de Investigación y Tecnología Animal, Instituto Valenciano de Investigaciones Agrarias, Apartado 187, 12400 Segorbe, Castellón Spain

**Keywords:** Pig, Score, Dry-matter, pH, Neonatal, Lactating, Diarrhea

## Abstract

**Background:**

Digestive disorders are one of the main health problems in suckling piglets. The correct visual identification of feces in suckling piglets is an important tool for the diagnosis of enteric diseases. The aim of the present observational study was to analyze different physicochemical parameters of the feces of suckling piglets aged 0 to 21 days: visual appearance (color and consistency), fecal dry matter (FDM) content and pH. A total of 482 fecal samples were collected and visually classified into six categories: meconium, colostrum stage feces and 4 further scores according to the degree of consistency: 0 = form; 1 = pasty; 2 = liquid; and 3 = watery feces. The percentage of FDM was estimated by two drying methods, oven and microwave, doing duplicates in each one to evaluate methods, and both were compared.

**Results:**

The most frequent colors of each feces category were dark green or dark brown for meconium; orange for colostrum; formed feces were mostly ocher and for the rest of the feces, the colors varied predominantly cream and ocher. Regarding FDM, liquid and watery categories had no statistically significant differences between them; meconium and colostrum feces FDM were not statistically different from pasty feces. The correlation coefficient between the FDM values of the duplicate analyses of the samples by both methods (oven and microwave) was very high (> 0.988). Importantly, no differences were found while comparing the results between both methods (*p* = 0.078), and the correlation coefficient between all samples analyzed with both methods was very high (> 0.98). Meconium was the only one that differed significantly from the rest in terms of pH.

**Conclusion:**

The physicochemical study of the feces of suckling piglets including color, FDM and pH allowed the establishment of an objective fecal score to characterize the stools in this age group.

**Supplementary Information:**

The online version contains supplementary material available at 10.1186/s40813-024-00417-2.

## Background

One of the main health problems in suckling piglets is digestive disorders, which result in economic losses, increased use of antibiotics, emergence of antimicrobial resistance and compromised animal welfare [1]. Sjölund et al., in 2014, reported that farms affected by 10% mortality rate due to neonatal diarrhea had economic losses of up to €134 per sow per year [2].

The correct identification of digestive problems in suckling piglets is based on behavior analysis, general condition of the animals and observation of the feces. Accurate observation and identification of the type of feces is important for the diagnosis of enteric diseases under productive conditions as well as for the development of experimental procedures. In 2011, Pedersen et al. demonstrated the difficulty of correctly classifying the consistency of feces from fattening pigs and suckling piglets among different observers [3–5].

The main challenge when analyzing feces from neonatal piglets is the small amount they excrete, sometimes less than a gram, along with the presence of undigested particles that hinder stool homogenization for analysis. In addition, in the first days of life, dark brown [6] or orange-colored feces with pasty or mucous consistency are excreted, which can be mistaken with pathological feces and are, in fact, physiological.

Different classifications of pig feces (fecal scores) have been used in studies to mainly evaluate the effect of different diets or supplements. For weaned pigs, some authors [7, 8] have used the same classification described by Marquardt et al. for suckling piglet, in which feces were classified into 4 categories: “0, normal; 1, soft feces; 2, mild diarrhea and 3, severe diarrhea”. In this method, category 1 is considered non-diarrheal [9]. Other studies in weaned pigs, such as those by Sun et al. [10] and Zhe et al. [11], maintained the classification in 4 categories, with some modifications, describing stools according to their consistency: 0, normal or solid; 1, pasty or semi-solid; 2, semi-liquid; and 3, liquid.

On the other hand, Pedersen, Toft and Strunz used, for growing and nursery pigs, the following classification into 4 categories: “score 1 = firm and shaped, score 2 = soft and shaped, score 3 = loose, and score 4 = watery” [4, 5]. Some studies have classified feces into only 2 categories: normal and diarrheal [12]. Metzler-Zebeli et al., in order to study the microbiome of the feces of lactating and weaned piglets, made a brief classification into 3 categories and considered color (dark brown (meconium), yellow, grey and brown) and consistency (balls, soft (normally shaped) and very soft (but still shaped) [6]. Other studies were based on 5 categories from hard and dry to watery stools [13, 14], and a study by Hancox et al. divided them into 7 categories [15]. Thus, there is no consensus for stool classification. In human medicine, there is also no single method of classification. Some authors divided human feces into 4 categories [16] and other protocols, such as the Bristol Stool Form Scale (BSFS) method, can divide them into 3 categories [17]. Other authors citing the BSFS divide feces into 7 categories of stool according to their consistency [18].

For fecal analysis it is advisable to study fecal dry matter (FDM). Pedersen et al. evaluated a microwave drying method for feces from weaned piglets and described the numerous and varied protocols for weaned pig stool evaluation used to date [19]. Therefore, there is no consensus on the standard protocol for fecal drying and FDM measurement.

For pH analysis in sow feces, Maes et al. [20] applied a pH measurement method described for horse feces [21] with some modifications. Additionally, differences in pH levels in the feces of suckling piglets have been described according to the existing pathology [22].

To date, there are no studies that have characterized and classified the feces of suckling piglets from birth to weaning based on physicochemical parameters and amount of FDM. Thus, the aim of the present observational study was to analyze different physicochemical parameters of the feces of suckling piglets aged from 0 to 21 days: visual appearance (color and consistency), FDM content and pH. For this purpose, some methods previously described for feces from pigs of other ages were used and modified, and two drying methods for FDM analysis (oven and microwave, MW) were compared. Based on such analyses, we propose a classification of the feces of suckling piglets that could be useful both for field work and for experimental studies.

## Methods

### Farm selection

The study was carried out on 4 commercial farms in northern Spain, from which written authorization was obtained from the owner of the animals. Two of the farms had Pietrain × (Landrace (LR) × Large White (LW)) piglets and the other two herds had Duroc × (LR × LW) ones. A total of 482 fecal samples were collected from individual suckling piglets from different litters, from 0 to 21 days of age (Table 1). At 1 week of life creep feed was offered to the piglets.

**Table 1:** Fecal samples collected per farm and number of litters and pigs represented in the sampling

Genetic background	Sows per farm (n)	Litters tested (n)	Sampled piglets (n)
Pietrain × (LR × LW)	2000	90	195
Pietrain × (LR × LW)	850	44	70
Duroc × (LR × LW)	1000	58	132
Duroc × (LR × LW)	3200	64	85
Total	7050	256	482

*LR* Landrace, *LW* Large white

Fecal samples were collected between January and April 2023 and between October 2023 and March 2024. Feces were collected in sealable jars when the piglets defecated in the morning. Inside the farrowing rooms, animals were observed for a variable period of time, between half an hour and 1 h, and feces were obtained when the animals defecated naturally or by stimulating defecation by massaging the anus and belly. Samples with insufficient weight for analysis (0.5 g for FDM and 0.3 g for pH) were discarded. Obtained feces were stored at 6 to 8 °C and analyzed within 24 h of collection.

### Classification and macroscopic analysis of stool

Feces were visually classified into six categories: meconium, colostrum stage feces and 4 further scores (0, 1, 2 and 3) according to the degree of consistency.

Meconium feces consisted of the first feces expelled by the newborn piglet, being dark in color and mucous in consistency. Feces derived from colostrum ingestion were expelled after meconium and were orange, pasty to mucous in consistency. Both categories were considered healthy feces. The remaining feces were categorized according to their degree of consistency with a modification of the criteria described by Marquardt et al. [9] and Pedersen et al. [3]: 0 = form (a healthy category); 1 = pasty; 2 = liquid (milky); and 3 = watery (dirty water) feces, which are associate with mortality, lethality or fatal diarrhea causing significant economic losses in suckling piglets [1, 23, 24]. Categories 2 and 3 were considered as diarrheic.

**Fig. 1:**
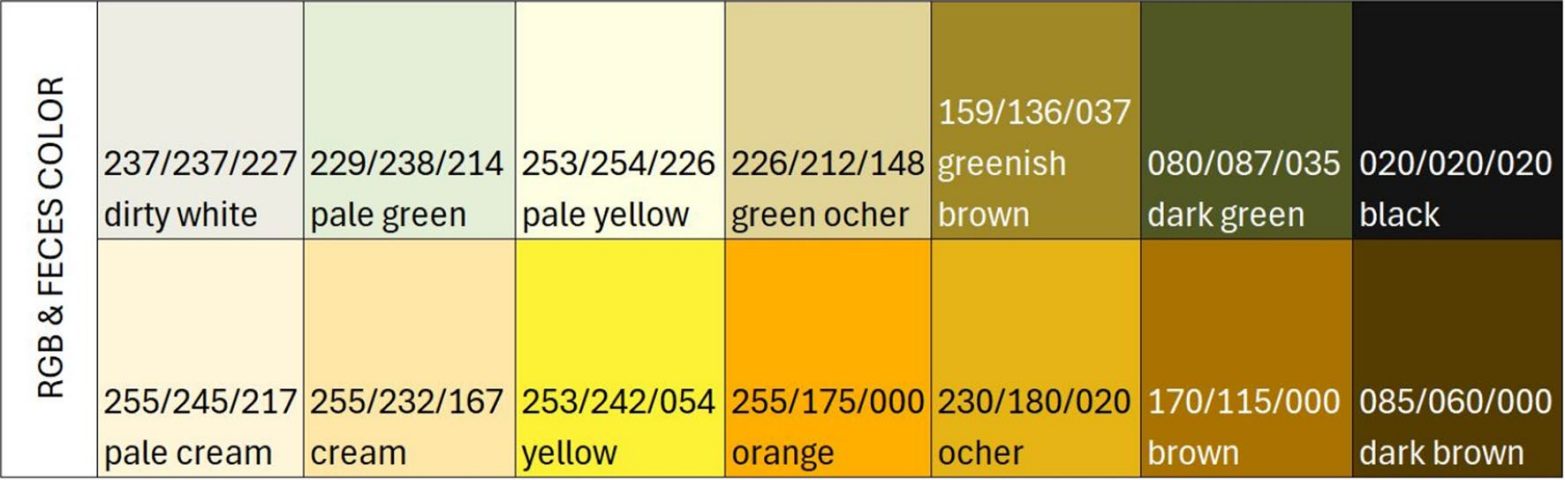
Classification of stool colors with their reference in the international RGB system

On the other hand, all feces were classified according to their color by calculating the % of each RGB (Red–Green–Blue) color scale for each feces category (Fig. 1).

### Physical–chemical analysis

The percentage of FDM was estimated by two drying methods: oven and microwave. In both cases the weight of the sample was measured on a Cobos HCT/D digital scale of 0.01 g of precision. Samples were handled and homogenized with a single-use wooden stick and fecal amounts weighing between 0.5 and 1.7 g were taken, depending on sample volume available. For drying, 10 mL glass beakers were used to avoid splashing. The beakers were tared at the beginning of the study, washed between samples with hot water and soap, rinsed with distilled water and dried in the oven where they were allowed to cool, being discarded and washed again if they differed from the reference weight by ± 0.04 g in the weighing.

Regarding the technique used for sample drying, on the one hand, special care was taken in washing and drying the containers, as well as to tare them correctly each time due to the small amount of sample to be dried in order to obtain repeatable results with duplicates. On the other hand, samples were homogenized by shaking them until homogeneity was observed by visual control.

Given the small amount of sample obtained from meconium or colostrum feces, it was prioritized to have enough sample for oven drying, which was used to establish the basic FDM in each category of samples. In the rest of the categories, the lactating feces, depending on the amount of sample available, feces were dried only in oven or only in microwave; when the amount of fecal material was 2 g or more, duplicates were taken, and the samples were dried in oven and microwave to compare the results with each drying system.

Oven drying was considered the standard method, based on drying of feed materials, and of choice in this study to establish the percentage of FDM of all collected feces. A universal forced-air stove, Memmert UF55, was used at 105 °C, following an adapted method from Ahn et al. [25] for the determination of dry matter in feed materials. The fan was set to 50%. Samples were dried for 1 h and 45 min, dried again for an additional 15 min, and further dried for another 5 min repeatedly until a constant weight was reached in two consecutive weightings (drying to constant weight), according to the criterion adapted from Pedersen et al. [19].

For the second drying method, a Samsung GW71E-S MW was used and the protocol described by Pedersen et al. [19] was followed. Fecal samples contained in 10 mL glass beakers were initially heated for 30 min at the lowest potency (120 W) followed by 10 min at medium potency (385 W).

The pH was measured with a PCE-228 digital pH-meter, dissolving the feces 1:3 in distilled water following the protocol described by Hydock et al. [21]. Between 0.3 and 0.4 g of feces were used. The laboratory analyses performed and the number of samples per category are indicated in Table 2.

**Table 2:** Summary of the number of fecal samples and type of analyses performed

Faecal category	Samples (n)	Type of analysis	pH analyses (n)	FDM analyses (n)			
				O_1	O_2	MW_1	MW_2
Meconium n = 84	25	Oven FDM	4	25	1		
	0	Microwave FDM					
	0	Oven and MW FDM					
	59	pH only	59				
Colostrum time feces n = 71	25	Oven FDM	4	25	2		
	0	Microwave FDM					
	0	Oven and MW FDM					
	46	pH only	46				
Consistent (score 0) n = 198	123	Oven FDM	50	123	24		
	40	Microwave FDM	4			40	39
	30	Oven and MW FDM	1	30	11	30	9
	5	pH only	5				
Pasty (score 1) n = 73	38	Oven FDM	17	38	7		
	10	Microwave FDM				10	6
	10	Oven and MW FDM	2	10	2	10	3
	15	pH only	15				
Liquid (score 2) n = 44	20	Oven FDM	10	20	8		
	5	Microwave FDM				5	5
	8	Oven and MW FDM	7	8	8	8	7
	11	pH only	11				
Watery (score 3) n = 12	4	Oven FDM	3	4	3		
	0	Microwave FDM					
	7	Oven and MW FDM	5	7	6	7	5
	1	pH only	1				
Total	482		244	290	72	110	74

n, Number of samples; FDM, Fecal dry matter; O_1, First analysis with oven; O_2, Second analysis with oven (duplicate); MW_1, First analysis with microwave; MW_2, Second analysis with microwave (duplicate)

### Statistical analyses

Excel 18.2311.1071.0 and R Studio 2023.06.0 + 421 were used for statistical analyses. A descriptive analysis was made by presenting type of feces in contingency tables, and a grouped bar chart was used for the color analysis.

To check the normality of the physicochemical variables (FDM and pH), the Agostino test for skewness and Bonett test for kurtosis of the data was used. Box plots were used to graphically observe the differences in pH and FDM between the six categories of stool described: meconium, colostrum stool and feces with scores 0, 1, 2 and 3. Analysis of variance (ANOVA) was used to compare results between categories/scores.

Two drying methods were used to assess FDM. The first step consisted of evaluating the homogeneity of the duplicates of both methods (oven and MW) to check whether one analysis per sample was sufficient or whether several analyses were necessary and obtain the average. The T-test for paired two-tail samples was used with a confidence level of 95%. The correlation coefficient between duplicates and the Lin’s Concordance Correlation Coefficient was also calculated; a scatter plot was used to represent these relationships between variables, where each point is equivalent to a sample with the value of the first analysis represented on the × axis and the value of the second analysis on the Y axis.

To analyze the differences between methods (oven versus MW), a T-test was used. Also, a specific graph to compare two methods, the Deming and Passing Bablock test, was used. The correlation coefficient between the values of the samples analyzed with both methods was calculated, and a prediction equation was estimated (using the R2 value as the fit goodness measure of the regression equation). A Bland–Altman plot of the agreement in FDM (%) was made for the comparison between both methods.

## Results

### Classification and macroscopic analysis of the feces

**Fig. 2:**
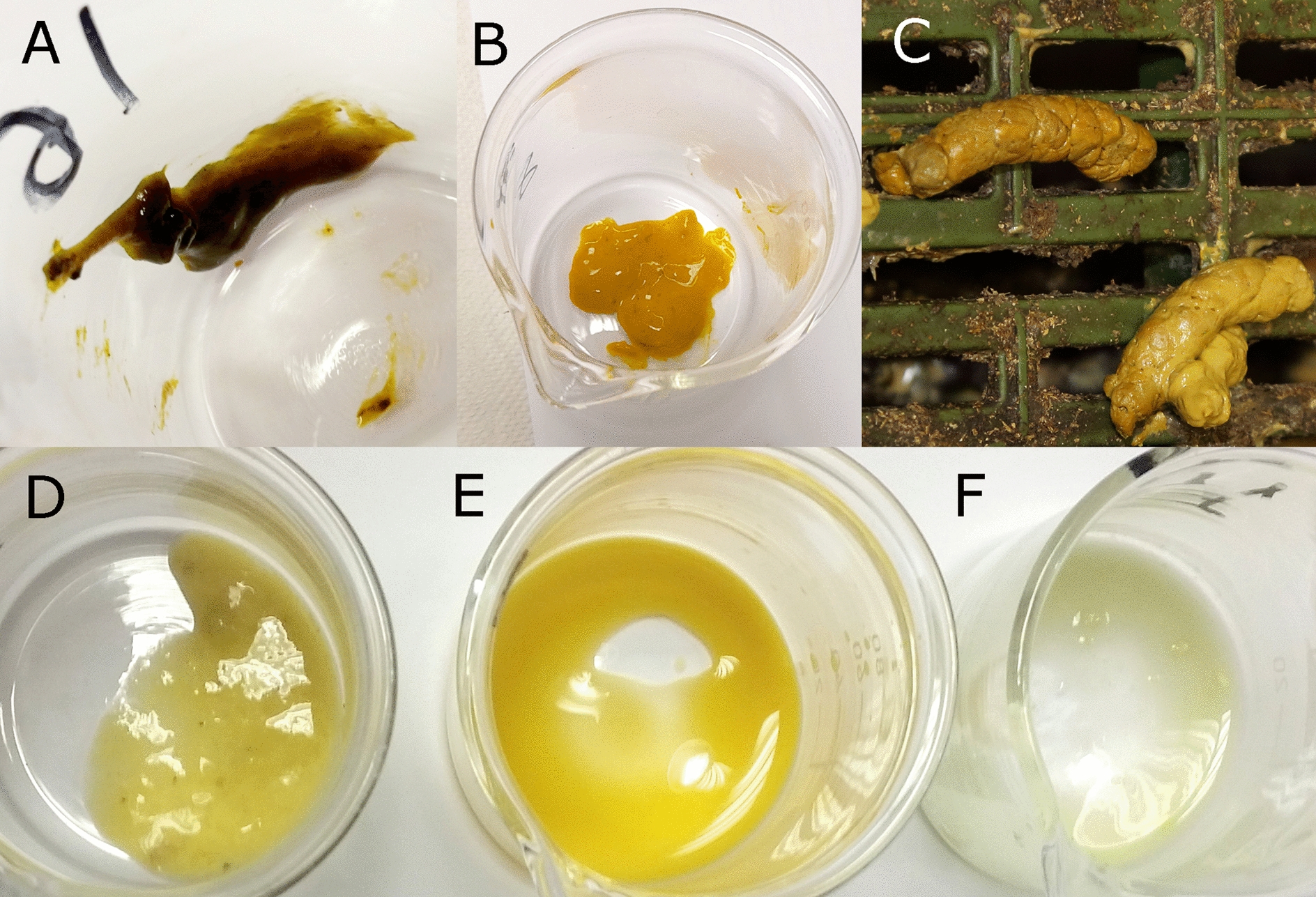
Images of visual feces consistency categories. Healthy feces (up): **A** Meconium. **B** Feces from the colostrum stage. **C** Score_0 form. And no healthy feces (down): **D** Score_1 pasty. **E** Score_2 liquid. **F** Score_3 watery

**Fig. 3:**
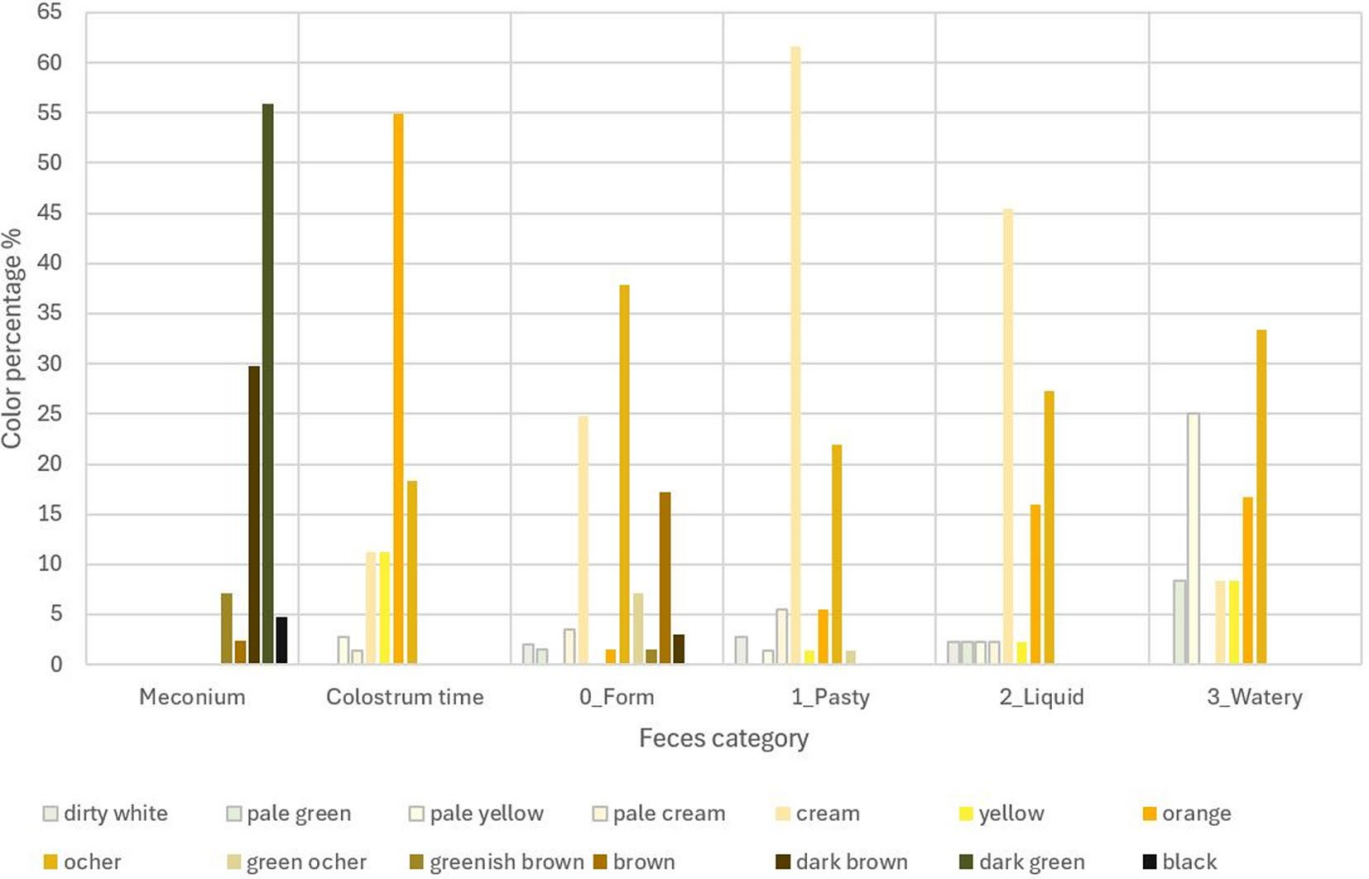
Percentage of colors for each stool category based on the RGB color scale

The consistency and coloration of each stool sample was recorded based on the different categories; the visual consistency of the feces is shown in Fig. 2 The percentage of samples of each color by type of feces is shown in Fig. 3.

The most frequent colors of meconium feces were dark green and dark brown. Orange was preponderant in the colostrum feces, followed to a lesser extent by ochre and yellow. In the formed feces cream, ocher and brown predominated. For the rest of the feces, the colors varied, with ocher, cream and orange being the most frequent ones in varying proportions depending on the degree of consistency. Cream color prevailed in the pasty and liquid feces, while ocher color predominated in the watery feces.

### Physicochemical analysis: FDM and pH

**Fig. 4:**
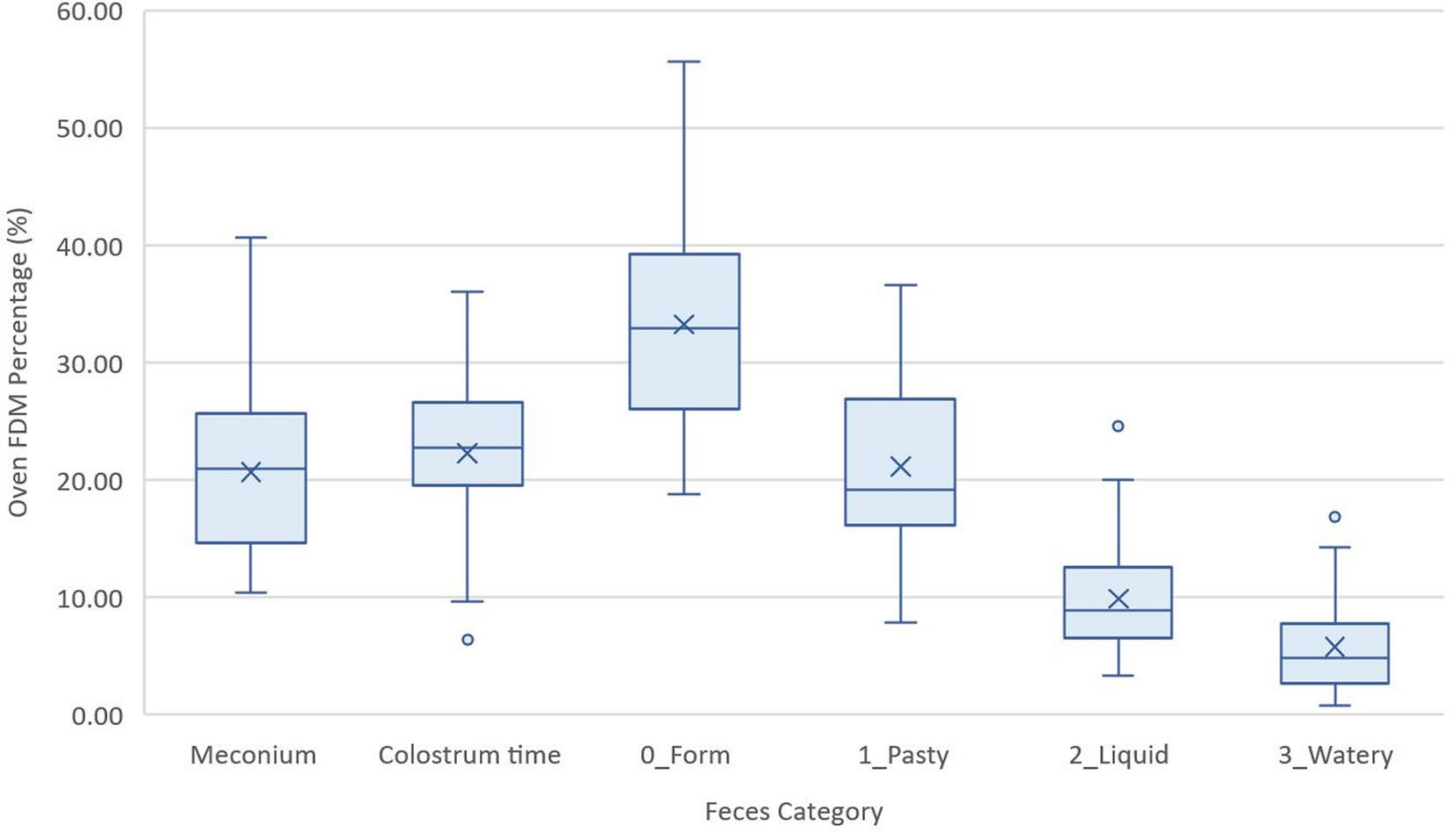
Percentage of FDM in oven for each of the six categories according to visual feces classification, with each median (line in the box) and mean (the crosses), the whiskers and the outliers

The percentage of FDM for each of the six categories is shown in Fig. 4. Table 3 shows the FDM values obtained for each type of feces.

**Table 3:** Summary oven FDM values (%) for each stool category

Stool category	Min	Q1	M	Mean	Q3	Max	V	ci	
Meconium	10.3	14.9	21.0	20.7	25.1	40.6	56.4	17.6	23.7
Colostrum time	6.4	19.7	22.8	22.2	26.2	36.0	48.8	19.5	25.0
0_Form	18.8	26.1	32.9	33.3	39.2	55.7	75.2	31.7	34.4
1_Pasty	7.8	16.3	19.2	21.1	26.6	36.6	51.2	19.2	23.0
2_Liquid	3.3	6.5	8.9	9.9	12.5	24.6	23.6	8.1	10.9
3_Watery	0.8	2.7	4.8	5.8	7.7	16.8	17.8	3.5	7.6

Min., Minimum; Q1, First quartile; M, Median; Q3, Third quartile; Max., Maximum; V, Variance; ci, Confidence interval

When analyzing the variable FDM, differences were observed between formed feces and all other categories (*p* < 0.001). Categories liquid and watery had no statistically significant differences between them, but both were significantly different from the others. The difference between non-diarrheic (pasty) and diarrheic feces (liquid and watery) was also statistically significant (*p* < 0.001). The pasty stools differed from the liquid and watery forms (*p* < 0.001), but not from the meconium and the excreta of the colostrum time.

**Fig. 5:**
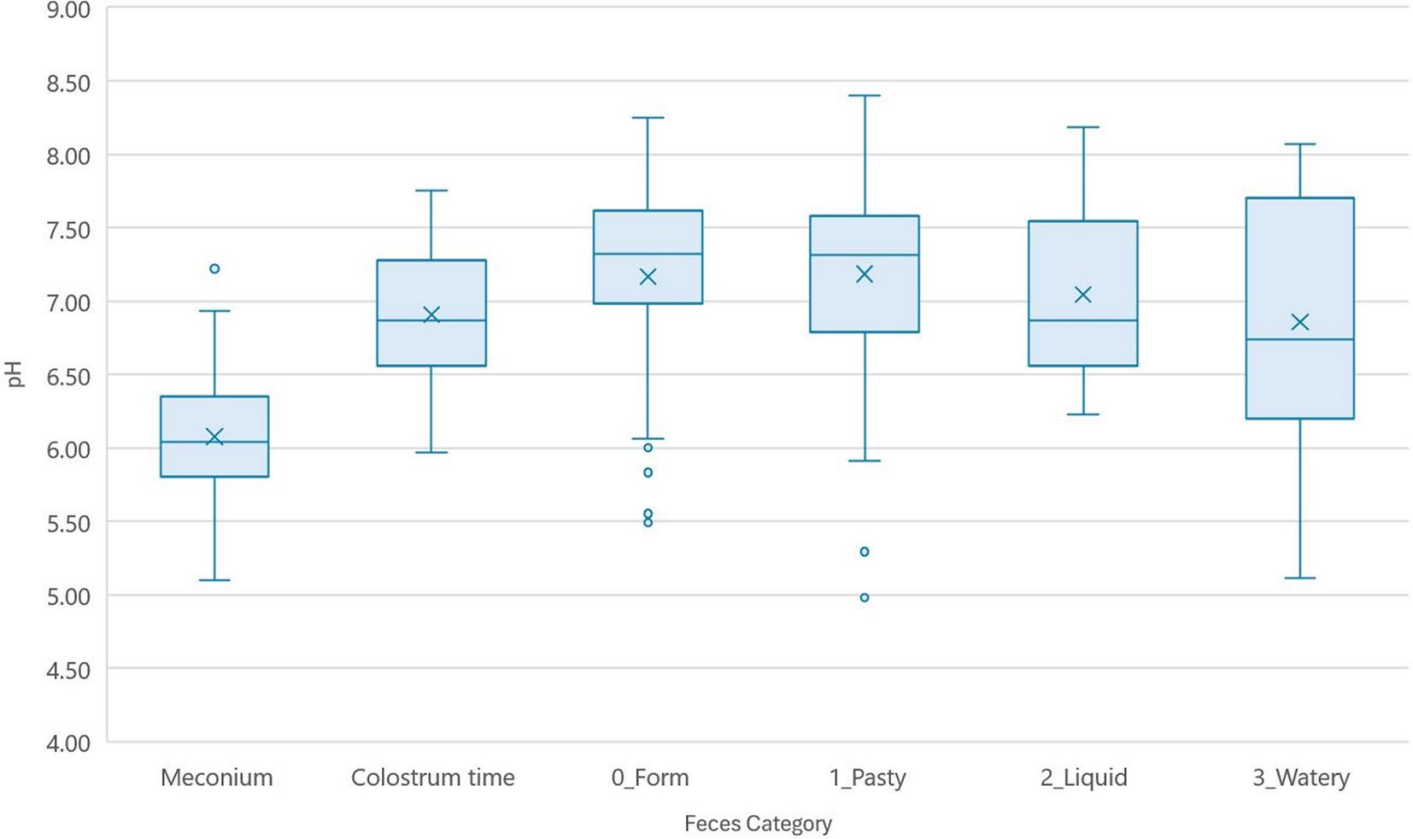
pH values for each of the six categories according to visual feces classification, with each median (line in the box) and mean (the crosses), the whiskers and the outliers

The pH values for each category are shown in Fig. 5. Table 4 shows the values obtained for each type of feces. In the ANOVA test for the pH, statistically significant differences were found between meconium and the other stool categories (*p* < 0.05).

**Table 4:** Summary pH values for each category of feces

Stool category	Min	Q1	M	Mean	Q3	Max	V	ci	
Meconium	5.97	5.81	6.04	6.08	6.35	7.22	0.20	5.96	6.19
Colostrum time	5.97	6.57	6.87	6.91	7.26	7.75	0.19	6.79	7.03
0_Form	5.49	7.02	7.32	7.16	7.61	8.25	0.41	7.10	7.41
1_Pasty	4.98	6.82	7.31	7.18	7.53	8.40	0.62	6.98	7.47
2_Liquid	6.23	6.57	6.87	7.04	7.53	8.18	0.36	6.98	7.47
3_Watery	5.11	6.48	6.74	6.86	7.44	8.07	0.93	6.11	7.60

Min., Minimum; Q1, First quartile; M, Median; Q3, Third quartile; Max., Maximum; V, Variance; ci, Confidence interval

### Comparison between drying methods for suckling piglet feces

#### Oven: homogeneity study

Oven data showed a normal distribution. The comparison between duplicates in the oven showed no significant differences (*p* > 0.065). Therefore, it was considered that feces of the suckling piglets were sufficiently homogeneous to perform a single analysis of each sample and obtain reliable FDM data.

**Fig. 6:**
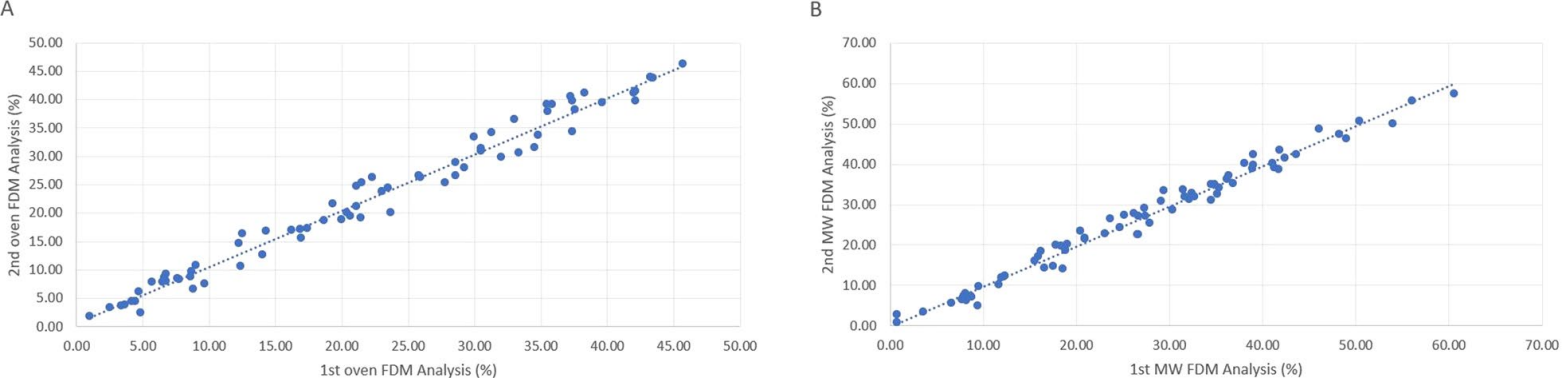
Scatters plots of FDM duplicates in oven and microwave. **A** Dispersion between samples analyzed in duplicate in the oven for FDM determination. **B** Dispersion of samples analyzed in duplicate in the microwave for FDM determination

The correlation coefficient between the values of the duplicate analyses of the samples was very high (> 0.988) (Fig. 6A). A bias correction factor of 0.999 was obtained in Lin's Concordance Correlation Coefficient (1 being perfect agreement).

#### Microwave: homogeneity study

Microwave data showed a normal distribution. No differences were observed between duplicates (*p* = 0.075), so it was considered that the stool samples analyzed were sufficiently homogeneous for this drying method. In Fig. 6B, the dispersion values of the moisture percentage of the first and second analysis are shown. The correlation coefficient between the duplicate values of all analyzed samples was very high (> 0.990). A bias correction factor of 0.999 was obtained in Lin's Concordance Correlation Coefficient.

### Comparison of oven and microwave drying methods for suckling pig feces for FDM determination

**Fig. 7:**
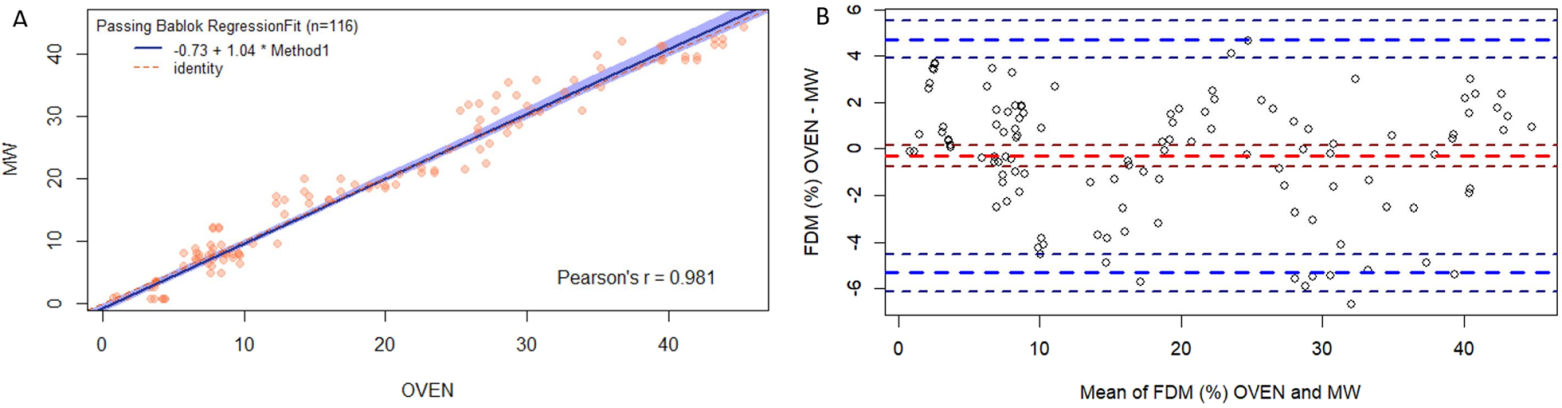
Graph analysis of Oven-Microwave comparison. **A** FDM agreement (%) for the results of the Oven-Microwave comparison. **B** Bland–Altman Plot of the FDM agreement (%) for the results of the Oven-Microwave comparison (red line = observed average agreement, blue line = 95% Limit for agreement of individual samples ± confidence interval)

No differences were found while comparing the results between both methods (*p* = 0.078). No samples of meconium or the colostrum stage were used in this comparison because of the low amount of excreted feces. The correlation coefficient between all samples analyzed with both methods was very high (> 0.98), as observed in the Deming and Passing Bablock analysis (Fig. 7A). The regression line includes the prediction (blue shadows) of microwave drying values from oven-drying values (MW values = − 0.73 + 1.04 * oven values). The value of slope was close to 1, with a value of Pearson’s correlation coefficient greater than 0.98. The confidence interval (ci) for the slope included 1, and the ci for the intercept included 0.

In the Bland–Altman plot (Fig. 7B) of the FDM agreement (%) for the comparative oven-MW, a great variation in the dry matter in the faeces was observed. Values can range from just over 1% of FDM to more than 40% (X axis). For the comparison between methods, the difference in results for the two techniques (Y axis) was mostly between + 4 to − 4 percentage points of FDM. The graph shows that the data between 10 and 40% of FDM are homogeneously distributed, while the data at the edges of the graph (X axis), both in diarrheal stools where FDM values were lower (< 10% FDM) and in dried stools where FDM had high values (> 40% FDM), are mostly above the average, over the red line.

## Discussion

Several studies [9, 10, 12] macroscopically classified feces from pigs of different ages to track the health status of the animals and to evaluate various treatments or experimental procedures. However, Pedersen et al. demonstrated the difficulty of correctly visually classifying the pig feces, highlighting the importance of a correct classification to assess the reduction of antibiotic use [4]. Furthermore, in 2013, Pedersen and Strunz described the challenge faced by farmers and veterinarians in visually identifying diarrhea in suckling piglets using images. These authors reported an increase of false positives in non-diarrheic animals, leading to an unnecessary increase in antibiotic consumption [5].

The objective of the present observational study was to perform a macroscopic and physicochemical characterization of suckling piglet feces from birth to weaning by establishing an objective classification considering visual consistency, FDM, pH and color. An accurate visual analysis of piglet stools should be able to facilitate a more precise diagnosis of digestive disorders. Such accuracy is expected to have a positive impact on the right use of antibiotics and, eventually, decrease their consumption.

One of the challenges encountered in the present study was the small quantity of feces obtained from most newborn piglets. This amount is often less than one gram, even barely 0.3 g, making subsequent analyses difficult. Therefore, a minimum of 0.5 g per sample was set to perform all FDM measurements. If this minimum was not exceeded, oven drying analyses were prioritized as the test of choice for FDM analysis.

Regarding feces color in newborns, meconium stools were almost black (dark green or dark brown) and stools in the colostrum stage displayed intense orange or yellowish color. Colostrum is produced by the sow on average up to 24 h after farrowing, but piglets can excrete feces generated by the colostrum intake up to 12–24 h after the dam stops producing colostrum. The change from the dark meconium feces to the orange feces of the colostrum time was not gradual but was a sudden change. Sometimes dark meconium feces were found inside the orange or yellowish colostrum stage feces, although in a perfectly differentiated manner one from the other. There was no single color for each type of stool and the range of colors for formed stools, considered normal, could vary from very whitish to dark brown or greenish, with ochre being the predominant color. These variations could be observed within the same litter, with the same feeding and environmental conditions and the same colors could be found in different days of age. In softer feces, paler colors such as cream predominated. Metzler-Zebeli et al. when analyzing the microbiome of the feces of piglets at days 2, 6, 13, 20, 27, 30 and 34 of life, weaned at day 28, observed statistically significant differences in the relationship of the microbiome with the color-consistency of the feces [6].

In numerous articles concerning diet or digestive pathology of piglets [7, 8, 10–15], reference was made to the visual appearance of the feces and their consistency. In the present study stools were classified into six categories, including meconium and stool from the colostrum stage. When studying FDM, statistically significant differences were observed between all established categories except between liquid and watery feces, although watery feces (dirty water aspect) are visually different than liquid stools (milky aspect). This may be due to the amount of undigested particles observed in watery feces as undigested coagulated milk or small particles of creep feed. Therefore, this macroscopic stool classification based on consistency may be practical for FDM analysis if we group together liquid and watery feces, making a classification in three consistency groups of lactating stools: form, pasty and a one single group of liquid feces (including watery ones), apart from meconium and colostrum feces.

Regarding the duplicates that were performed for analyzing FDM, both methods oven and MW offered equivalent results, so, a single analysis would be considered sufficient to calculate the FDM value of the feces However, in the same way as in weaned piglet stools, the microwave is a much faster technique to get results and it is considered a more practical and economical method [19]; therefore, it was concluded that with the small amount of sample available, the result of a single analysis was robust enough.

For pH analyses, pH paper strips were initially tested, but were discarded because the color of the feces masked the measurements (data not shown). The 1:3 dilution method in distilled water [21] allowed to have a sufficient sample to be able to measure this parameter with a digital pH-meter. The disadvantages of this method include the difficulty of using the digital pH-meter under field conditions due to its fragility and the need to clean it very carefully, rinse it with distilled water, and dry it after each sample. For healthy feces (meconium, colostrum or formed feces) pH values ranged from slightly acidic to neutral pH, with significant differences between categories, probably due to the changes that occur in the diet after birth (ingestion of colostrum and then mother's milk). No statistically significant differences were found between the rest of the categories (pasty, liquid and watery), all with values close to neutral pH. Luppi [22] described differences in pH levels in the feces of neonatal piglets according to the existing pathology: alkaline pH for *E. coli* infections and coccidiosis or acid pH for viral infections. In the present study, no microbiological studies were carried out on feces considered diarrheic; however, in the absence of these studies, pH did not appear to be a discriminating value when classifying different types of consistency of suckling pig feces, including those cases with diarrhea.

## Conclusions

Visual inspection continues to be the quickest and most useful approach, along with other clinical signs, to assess the severity of a digestive process in swine production. In this study, the feces of lactating animals, in addition to meconium and colostrum feces, have been characterized. The type of feces during the first 24 h of life can be confused with pathological feces due to their color and consistency. However, it has been possible to establish a range of colors for each of the six categories studied, meconium, colostrum stool and scores 0, 1, 2 and 3 according to their visual appearance. The FDM analyses correlated well with the different categories proposed except between liquid and watery which can be grouped together. pH values only showed differences between meconium and the rest of fecal sample types. Finally, the analyses confirmed the microwave drying technique, compared to oven drying, as the easiest and fastest test for FDM analysis in suckling piglets.

## Supplementary Information

Additional file 1.

## Data Availability

Data can be found in the additional file.

## Abbreviations

ANOVA: Analysis of variance
CEGECO: Compañía General de Compras Agropecuarias S.L.U.
ci: Confidence interval
CReSA: Centre de Recerca en Sanitat Animal
FDM: Fecal dry matter
IRTA: Institute of Agrifood Research and Technology
LR: Landrace
LW: Large White
M: Median
Max.: Maximum
Min.: Minimum
MW: Microwave
MW_1: First microwave analysis
MW_2: Second microwave analysis (duplicate)
n: Number of samples
O: Oven
O_1: First oven analysis
O_2: Second oven analysis (duplicate)
Q1: First quartile
Q3: Third quartile
UAB: Universitat Autònoma de Barcelona
V: Variance
WOAH: World Organisation for Animal Health
